# PRG-1 relieves pain and depressive-like behaviors in rats of bone cancer pain by regulation of dendritic spine in hippocampus

**DOI:** 10.7150/ijbs.59032

**Published:** 2021-09-21

**Authors:** Xingfeng Liu, Zhuo Xie, Site Li, Jingxin He, Song Cao, Zhi Xiao

**Affiliations:** 1 Guizhou Key Laboratory of Brain Science, Zunyi Medical University, Zunyi 563000, China.; 2 Guizhou Key Laboratory of Anesthesia and Organ Protection, Zunyi Medical University, Zunyi 563000, China.; 3 Graduate School, Zunyi Medical University, Zunyi 563000, China.; 4 Department of Pain Medicine, Affiliated Hospital of Zunyi Medical University, Zunyi, 563000, China.

**Keywords:** bone cancer pain, PRG-1, P2X_7_ receptor, depression, dendritic spine

## Abstract

**Rationale:** Pain and depression, which tend to occur simultaneously and share some common neural circuits and neurotransmitters, are highly prevalent complication in patients with advanced cancer. Exploring the underlying mechanisms is the cornerstone to prevent the comorbidity of chronic pain and depression in cancer patients. Plasticity-related gene 1 (PRG-1) protein regulates synaptic plasticity and brain functional reorganization during neuronal development or after cerebral lesion. Purinergic P2X7 receptor has been proposed as a therapeutic target for various pain and neurological disorders like depression in rodents. In this study, we investigated the roles of PRG-1 in the hippocampus in the comorbidity of pain and depressive-like behaviors in rats with bone cancer pain (BCP).

**Methods:** The bone cancer pain rat model was established by intra-tibial cell inoculation of SHZ-88 mammary gland carcinoma cells. The animal pain behaviors were assessed by measuring the thermal withdrawal latency values by using radiant heat stimulation and mechanical withdrawal threshold by using electronic von Frey anesthesiometer, and depressive-like behavior was assessed by sucrose preference test and forced swim test. Alterations in the expression levels of PRG-1 and P2X7 receptor in hippocampus were separately detected by using western blot, immunofluorescence and immunohistochemistry analysis. The effects of intra-hippocampal injection of FTY720 (a PRG-1/PP2A interaction activator), PRG-1 overexpression or intra-hippocampal injection of A438079 (a selective competitive P2X_7_ receptor antagonist) were also observed.

**Results:** Carcinoma intra-tibia injection caused thermal hyperalgesia, mechanical allodynia and depressive-like behaviors in rats, and also induced the deactivation of neurons and dendritic spine structural anomalies in the hippocampus. Western blot, immunofluorescence and immunohistochemistry analysis showed an increased expression of PRG-1 and P2X_7_ receptor in the hippocampus of BCP rats. Intra-hippocampal injection of FTY720 or A438079 attenuated both pain and depressive-like behaviors. Furthermore, overexpression of PRG-1 in hippocampus has similar analgesic efficacy to FTY720. In addition, they rescued neuron deactivation and dendritic spine anomalies.

**Conclusion**: The results suggest that both PRG-1 and P2X_7_ receptor in the hippocampus play important roles in the development of pain and depressive-like behaviors in bone cancer condition in rats by dendritic spine regulation via P2X_7_R/PRG-1/PP2A pathway.

## Introduction

Recent advances in cancer diagnosis and treatments mean that 50% cancer patients can expect to survive the disease for 10 or more years 1, whereas depression, a feeling of hopelessness, anhedonia, decreased cognitive function and impaired everyday activity are common to terminal patients with cancer, and cancer related chronic pain deteriorates depression-like behaviors 2. Meanwhile, pain and depression are two common complications of advanced cancer, but often had an insufficient emphasis, influencing quality of life, treatment compliance, and cancer survival 3, 4. Depression affects up to 20% of patients with cancer, compared with the figure of 5% for prevalence in the general population in 2017 5. Thus, the management of pain in patients with cancer is a challenge and exploring the mechanisms underlying the comorbidity of chronic pain and depression in cancer patients is the foundation for pain and depression comorbidity control.

Around 30-50% cancers, particularly malignant metastases in bone, and almost all patients with advanced cancer, will experience persistent pain during the course of their disease, and pain intensity appears to increase as cancer progresses 6-9. Local factors including direct damage to sensory nerve fibres, infiltration of nerve roots by cancer cells and algogenic biological agents within the microenvironment of the tumor bring about central sensitization of dorsal horn neurons, characterized by neurochemical reorganization with persistent cancer pain 6, 8, 10-12. After integration and modulation at the spinal level, the enhanced pain messages are transmitted to the brain, where the final pain experience is generated 13. Finally, pain alters function and activity in the descending controls, which relay information from higher brain centers via the midbrain and brainstem to the spinal cord, ongoing and evoked pain and comorbidities 13, 14. The persistent barrage of neural signals delivered by primary nociceptors induces structural and functional changes in the dorsal horn of the spinal cord, and this neural plasticity facilitates the development of persistent central sensitization, and consequently promotes bone cancer pain (BCP) 10, but the underlying mechanism is still not clear.

The hippocampus is one of the key brain regions in pain signals modulating 15, particularly with respect to the affective aspect of pain perception 16, 17 and abnormal emotional processes, including anxiety and depression 18. Accumulating studies have suggested that chronic pain arising from peripheral nerve injury induces alterations in different areas of the brain including impaired hippocampal mossy fiber-CA3 synaptic plasticity and DG neurogenesis 19-21. The hippocampus functional connectivity networks were disturbed under chronic pain condition by an fRMI study 22, 23. In addition, many studies found that neuropathic pain rats exhibited structural and functional alterations in the hippocampus after peripheral nerve injury 24, 25, including hippocampal glutamatergic synapses loss 26. Studies on chronic pain in patients and animal models have shown hippocampal volume loss 27, interfered synaptic plasticity and neurogenesis 15, 24, 28, 29. Consistent with these, several experimental studies have found that direct hippocampal manipulation alters nociceptive behavior. Activation of the dorsal hippocampus reverses neuropathic pain through local excitatory and opioidergic mechanisms affecting dorsal hippocampus functional connectivity 30. Direct injection of the local anesthetic like lidocaine to the dentate gyrus (DG) region of the hippocampus produces analgesia 31. Neuropathic pain induced-depressive-like symptoms and hippocampal plasticity are dependent on hippocampal TNFR1 (tumor necrosis factor receptor 1) signaling. TNFR1(-/-) mice did not change hippocampal neurogenesis and plasticity after injury nor develop depressive-like symptoms 29. However, the underlying mechanism of hippocampus regulation of pain or depression is still not clear. Therefore, it is necessary to explore the hippocampal cellular and molecular changes during chronic pain conditions.

Plasticity-related gene 1 (PRG-1) is the prototype of the family of plasticity-related genes (PRGs) which are called lipid phosphate phosphatase (LPP)-related proteins (LPPRs) according to nomenclature rules based on their structural similarity to the LPP family members 32, also termed LPPR-4 33. Hippocampal PRG-1 plays an important role in the maintenance of synaptic homeostasis by modulating glutamatergic junction 34 and intracellular protein phosphatase 2A (PP2A)/β1-Integrin signaling 35. The glutamatergic junction signaling pathway involves lysophosphatidic acid (LPA) via presynaptic LPA2 receptors and PRG-1 from the postsynaptic side 34. Deletion of PRG-1 in mice leads to epileptic seizures and augmentation of excitatory postsynaptic currents (EPSCs), which further confirmed the important role of PRG-1 in modulation of hippocampal excitability 34. The PRG-1 intracellular PP2A/β1-Integrin signaling could affect synaptic plasticity and memory formation via interaction of its cytoplasmic calmodulin-binding domain (CBD) 35, 36. The numbers of the spine in hippocampal CA1 sr, CA1 so, and moDG regions revealed significantly lower densities in PRG-1-deficient mice compared with wild type (WT) littermate controls on P12, P19 in mice 35. In study on human tissue, PRG-1 was found differentially regulated in the substantia nigra of clinically and histologically confirmed epileptic seizures and Parkinson' s disease patients 37. At present, studies on PRGs family mainly focus on neuropsychiatric diseases and nerve injuries such as epilepsy 34, 38, 39, schizophrenia 40, 41, memory disorders 35, nerve trauma 42, 43, sensory discrimination deficit 44, 45, etc. Besides, PRG-1 may be involved in cancerogenesis because the target role of LPA in cancer 33, 46. However, whether PRG-1 or PRGs family play a role in pain or depressive-like behaviors has not been investigated.

P2X_7_ receptor, a kind of adenosine triphosphate (ATP) ligand gated ion channel receptor subtype, was a research spotlight in recent years due to the particularity of its molecular structure. P2X_7_Rs have been proposed as therapeutic targets for various inflammation, pain, tumor and neurological disorders 47-51 as well as for autism and depression-like behavior in mice 52, 53. The activation of spinal and supraspinal P2X_7_ receptors plays a key role in the occurrence and development mechanisms of various kinds of acute or chronic pain 54-56, including neuropathic pain and inflammatory pain 49, while the exact role of P2X_7_ receptor in BCP still remains controversial. The activation of P2X_7_ receptor is involved in development process of BCP by the mechanism of descending facilitatory by Huang et al. 57. While P2X_7_ receptor plays an analgesic effect on BCP by Hansen et al. 58 and Li et al. 54. In the tumor microenvironment, ATP is released to form a local high concentration ATP environment, which activates P2X receptors and causing the influx of Ca^2+^ in cells 59. Repeatedly relatively high concentrations of ATP stimulate the formation of membrane pore depended on P2X_7_ receptor, which is closely related to the formation of mechanical touch induced pain in mice, arthritis, and pain in patients after mastectomy 60. Large numbers of Ca^2+^ influx activate a variety of downstream cell signal transduction pathways that excite nociceptive neurons, thereby increasing their excitability and resulting in pain sensation 59, 61, 62. Rett syndrome (RTT), an early-onset neurodevelopmental disorder, resulted in accumulation of P2X7R-expressing monocytes and macrophages located at the edge of the cerebral cortex, while P2X7R deficiency or pharmacological block restored cortical dendritic spine plasticity, and ameliorated social behavioral defects of RTT mice 63.

In this study, for the first time, we found that hippocampal PRG-1 activation is involved in the regulation of BCP, and there is partial co-localization of hippocampal PRG-1 and P2X_7_ receptors, which suggested there existed a brand new pain modulation signaling pathway. Additionally, we identified that PRG-1 is involved in BCP and depressive-like behaviors by regulating dendritic spine development in hippocampus via P2X7R/PRG-1/PP2A signaling. Based on the results of this study, a new pathway modulating BCP was explored, which is helpful to expand a novel analgesia and depression relief strategies targeting PRG-1.

## Methods

### Animals

Pathogen-free, female Sprague-Dawley rats weighing 210±10 g (n = 162) were purchased from the Tianqin Biotechnology Co. Ltd. (license number: SCXK (xiang) 2019-0014; Changsha, China) and used for all experiments. Rats were housed in a temperature (23 ± 2 °C) and humidity (55 ± 5%) -controlled environment, with a 12-h light/dark cycle (8:00 a.m. - 8:00 p.m.) and free access to food and water. All experimental procedures were in accordance with the guidelines of the Ethical Committee of the International Association for the Study of Pain 64 and ethically approved by the Animal Care Ethics Committee of Zunyi Medical University. Try to minimize the number of animals used and reduce their suffering from experimental procedures.

There were 54 rats in the first part of the experiment: normal group (n = 18), sham group (n = 18) and BCP group (n = 18) for behaviors test, and 6 rats in each group were used for western blots and RT-PCR, 6 rats in each group were used for Golgi staining and the rest 6 rats were used for nissl staining and immunofluorescence. There were 48 rats in the second part of the experiment: BCP +FTY720 group (n = 18), BCP +A438079 group (n = 18), BCP +BzATP group (n = 6) and BCP +Okadaic Acid (OA) group (n = 6). There were 60 rats in the third part of the experiment: BCP +PRG-1 overexpression (OE) group (n = 12), BCP +PRG-1 knock down (KD) group (n = 12), BCP +P2X_7_R KD group (n = 12), BCP +PRG-1 OE +BzATP group (n = 6), BCP +PRG-1 KD + FTY720 group (n = 6), BCP +PRG-1 KD +A438079 group (n = 6), and BCP +P2X_7_R KD+ FTY720 group (n = 6). Cutaneous sensitivity to thermal and mechanical stimulation was measured before surgery and every 5-7 days after surgery for 6 weeks. Sucrose preference test was examined every week and forced swim test was performed on postoperative day 20. BCP Rats without depression-like behavior were not included in the statistics. On postoperative day 45, all rats were killed and the hippocampus was harvested for further analyses. The experimenter doing behavioral tests was blinded to the groups of animals.

### Animal model of bone cancer pain

All rats were assessed for their baseline thresholds to mechanical and thermal stimuli before surgery. Only the rats with normal baseline responses were used.

BCP model was established as described previously 65. In brief, the rats were anesthetized with pentobarbital sodium (50 mg/kg body weight, intraperitoneal, i.p.), followed by a 1.5-cm incision on the right leg of the rat to expose the tibia. 20 μL of SHZ-88 carcinoma cells (10^7^ cells/mL), purchased commercially from the Shanghai Cell Bank of the Chinese Academy of Sciences (Shanghai, China), was slowly injected into the intramedullary cavity of the right tibia. The injection site was immediately sealed with sterile bone wax, and the incision wound was treated with penicillin and layered sutured. The sham group was injected with boiled cells (20 min/ 95 °C) instead. The day of carcinoma cells inoculation is day 0.

### Bone radiological detection

Tibia radiological detection was performed on day 28 after intra-tibia injection of SHZ-88 to determine bone destruction. After anesthesia with pentobarbital sodium (50 mg/kg body weight, i.p.), the right hind limb of rats was irradiated with an intraoral X-ray source under the condition of 0.32 s at 60 kV, 7 mA (Kodak, 2100, Carestream Health, Inc.). And the radiographs of contralateral tibia were taken as control.

### Hematoxylin-eosin (HE) staining

Histological staining was assayed as described 66 with little modification. Rat tibia was separated, rinsed in normal saline, and fixed in 4% paraformaldehyde (PFA) overnight, followed by decalcification in EDTA decalcifying solution (Solarbio) in 4 °C for 2 weeks until easily penetrated through by a needle. Dehydration, transparency and paraffin embedding were operated as standard protocols. 5 μm sections were sliced using paraffin microtome (Leica RM2235, Germany) and pasted on gelatin slides. The sections were stained with hematoxylin and eosin staining kit (Beyotime) according to the manufacturer's protocol. The mounted slides were observed under light microscope (Leica, Germany).

### Implantation of hippocampal cannula and microinjection procedures

After anesthesia with pentobarbital sodium (50 mg/kg body weight, i.p.), the rats were placed on a stereotaxic frame (RWD Life Science, China). The surface of skull was exposed. The virus expression vector LV-Plppr4, LV-Plppr4-RNAi or LV-P2X7R-RNAi (genechem, Shanghai) was slowly injected (200 nl/min) bilaterally into the hippocampus area through a fine glass pipette by microsyringe pump (RWD Life Science, China) a week before BCP operation. The pipette remained in place for 10 min to ensure complete diffusion of the virus and then slowly removed 67. The stereotaxic coordinates of hippocampus were AP = -3.72 mm, ML = ±2.0 mm, and DV = -3.0 mm, according to the rat brain atlas 68.

For drug treatment experiments, a guide cannula [0.48 mm outside diameter (O.D.); 0.34 mm inner diameter (I.D.)] was implanted into the hippocampus and fixed to the skull of rats. For micro-injection, rats were inserted with an injection cannula (0.3 mm O.D.; 0.5 mm longer than the guide cannula) into the guide cannula. A total volume of 0.5 μL was slowly injected, and the injector kept in place for 10 min to allow complete diffusion of the drug before slowly withdrawn. Microinjection sites were checked by histological examination 54.

### Behavioral assessment

#### Inclined plane test

The functional deficit of motor, including muscular strength and proprioception, was quantitated using an inclined plane as reported by Rivlin et al. 69. The rats were placed crosswise on an inclined plane which can be adjusted to provide a slope of varying grade. The initial angle of the inclined plane was 25, and the angle was increased slowly by 5 degrees. The maximum angle of the plane at which the rats can maintain its position for 5 s without falling was recorded. The average values from the measurements of five times for each rat were defined as the inclined plane degree 54.

#### Radiant heat test

Thermal hyperalgesia was tested in rats as reported by Hargreaves et al. 70 previously to evaluate thermal withdrawal latency (TWL). Each rat was placed in an individual Plexiglass house, allowed to acclimate for 30 min until they settled down. 52 ± 0.2 °C radiant heat (50 W, 8 V bulb) was applied to the plantar surface of the hind paw with the plantar radiant heat instrument (IITC Life Science Instruments, USA). The latency period was recorded until the removal of the paw (including lifting, licking, flicking, shook or jumping). The cut-off limit was set to 60 s. Each hind paw was measured for five times at 5-min intervals 54.

#### The electronic von Frey meter test

Electronic pressure meter test described by Vivancos GG et al. 71 was carried out to evaluate mechanical withdrawal threshold (MWT). Each rat was placed in an individual plastic chamber for 30 min until they calm down. Mechanical allodynia of rat hind paw was measured with the electronic von Frey anesthesiometer (IITC Life Science Instruments, USA). The polypropylene tip was applied to the plantar surface of the hind paw with a gradual increase in pressure until a functional response (including lifting, licking, flicking, shook or jumping). The threshold pressure was recorded. Each hind paw was measured for five times at 5-min intervals.

#### Sucrose preference test

Rats were trained to the presence of 2 identical drinking bottles, 1 containing 1% (w/v) sucrose solution and the other containing pure water before the experiment. After 24-h water deprivation, the rat was fed in a solitary cage, given a bottle of 1% (w/v) sucrose solution and a bottle of pure water for 3h. The position of the two bottles was swapped every 30 min to avoid position preference. The volume of sucrose solution and pure water intake were measured. The ratio of sucrose solution intake to the total liquid intake was calculated as sucrose preference (%). The decrease in sucrose preference (%) indicates an anhedonia for rats, which is a manifestation of depressive-like behavior 4.

#### Forced swim test

On the first day, rats were placed in a transparent cylindrical acrylic container (a height of 60 cm and a diameter of 20 cm) filled with tap water (22-24 °C, water height 45 cm) for 15 min swimming acclimation. On the second day, rats were forced to swim for 6 min under the same conditions. The whole experiment was recorded with a camera. The immobility time of the hind limbs was counted in the last 4 minutes. Increased ratio of immobility time was used to assess despair and hopeless behavior of the rats.

### Perfusion, immunohistochemistry and immunofluorescence

Rats were deeply anesthetized with pentobarbital sodium (50 mg/kg body weight, i.p.) for the perfusion of normal saline (NS) through the left ventricle followed by 4% PFA. The brains were harvested, post-fixed in 4% PFA for 4-6 h at 4 °C, and cryoprotected in 30% sucrose at 4 °C until they sank. The brains were coronally cut at a thickness of 30 μm on a cryostat (Leica CM 1950, Germany).

For immunofluorescence, the sections were blocked and permeabilized, and then incubated with goat anti-rat P2X_7_ receptors antibody (1:400, abcam, USA) or rabbit anti-rat PRG-1 antibody (1:500, synaptic system). Sections were incubated in secondary antibody conjugated with CY3 donkey anti-rabbit (1:1000, abcam) and Alexa 488 donkey anti-goat (1:1000, abcam). The captured images were observed under a fluorescence microscope (Leica, Germany).

### Co-immunoprecipitation (CoIP) and western blot (WB)

The whole hippocampus was dissected and mechanically homogenized, then lysed in appropriate volume of radioimmunoprecipitation assay (RIPA) lysis buffer (Beyotime, China) containing phenylmethanesulfonyl fluoride (PMSF) (YEASEN) for 1 h on ice, and cleared by centrifugation for 10 min at 15000 g at 4 °C. Protein concentrations of the lysate were determined using Bradford reagent (Bio-Rad, Hercules, CA, USA).

**For co-immunoprecipitation,** the lysate was precleared with agarose slurry, and incubated with PRG-1 antibody (1:100, synaptic system), then pulled down by Protein G agarose resin (absin). Finally, beads were suspended with appropriate amount of lysis buffer and analyzed by western blot.

**For western blot**, tissue lysates or immunoprecipitated samples were separated by 10% sodium dodecyl sulfate-polyacrylamide gel electrophoresis (SDS-PAGE) and transferred onto polyvinylidene fluoride (PVDF) membrane (Biosharp). Then membranes were incubated with first antibodies, including PRG-1 (1:3000, synaptic system), P2X_7_R (1:1000, abcam), PPP2R2A (1:1000, Cell Signaling), ß-actin (1:5000, MP Biomedicals), and horseradish peroxidase (HRPO)-conjugated secondary antibodies (1:5000, dianova). Finally, membranes were developed by ECL (EpiZyme scientific).

### Nissl staining

Nissl staining was used for the staining of nissl body in the cytoplasm of neurons. The frozen section was washed with distilled water after 4% PFA fixing for 20min. The sections were then stained with nissl staining solution (Beyotime) for 3- 10min according to the dyeing results and requirements. The sections were washed twice with distilled water and then 70% ethanol. Finally, the changes of nissl bodies were observed under a microscope 72. For quantification, clear and intact neural cells with nissl bodies uniformly distributed around the nuclei in the hippocampal CA1 and DG regions were counted in a blinded manner. Then the percentage of nissl positive neurons to the total number of neurons was calculated 73.

### Golgi-Cox staining and dendritic synapse quantification

Golgi staining was used to examine synaptic plasticity. After anesthesia (50 mg/kg body weight, i.p.), the brains of rats were removed and immediately stained by Hito Golgi-Cox OptimStain^TM^ Kit (Hitobiotec, USA) according to the manufacturer's protocol and then photographed under a light microscope. Spines were defined as dendritic protrusions and were manually counted along a selected dendritic segment using ImageJ 6.0 software (NIH, USA) 74, 75. In brain sections we focus our analyses on dendrites of three areas: (1) the middle molecular layer of dentate gyri (moDG) granule cells; (2) stratum radiatum (sr, apical dendrites) and (3) stratum oriens (so, basal dendrites) of pyramidal neurons of the CA1 region. For each group around 1000 spines or at least 1000 µm dendritic length were measured. For each group, the density of dendritic spines, that is, the number of spines per µm dendrite was calculated 35. Analysis was performed in a blinded manner.

### RNA extraction and quantitative reverse‐transcription polymerase chain reaction (qRT-PCR)

The entire hippocampus was collected and RNA was extracted using TRIzol and reverse‐transcribed into cDNA using the PrimeScript™ RT reagent Kit (Takara, Japan). The differential expression of each gene was analyzed following polymerase chain reaction (PCR) using the TB Green® Premix Ex Taq™ II (Takara). The relative expression was defined as *F*=2^-ΔΔct^. The primers were synthesized by Sangon (Shanghai, China) and listed in Table 1.

### Statistical analysis

Data were processed with the GraphPad Prism (version 8.0.2; GraphPad Software, Inc., USA). All results are expressed as the mean ± SEM (standard error of mean) if not indicated otherwise. Data statistical analysis was done using two-tailed unpaired student t-test for comparing two groups with normal distributed data or a Mann-Whithnes-U test for comparing two groups containing nonparametric distributed data. Data normalized to control values (rendering control values as 1) were calculated using a one-sample t test. One-way ANOVA with Bonferroni correction for multiple comparing groups containing normal distributed data or a Kruskal-Wallis test with a Dunn's multiple comparisons test for multiple comparison of groups containing nonparametric distributed data. Normal distribution was assessed using the Kolmogorov-Smirnov-Test. Statistical significance was determined with an overall significance level of *p*<0.05 (n.s. for *p*>0.05, *p<0.05, **p<0.01, ***p<0.001).

## Results

### Evaluation of bone destruction in BCP model

**Figure 1:**
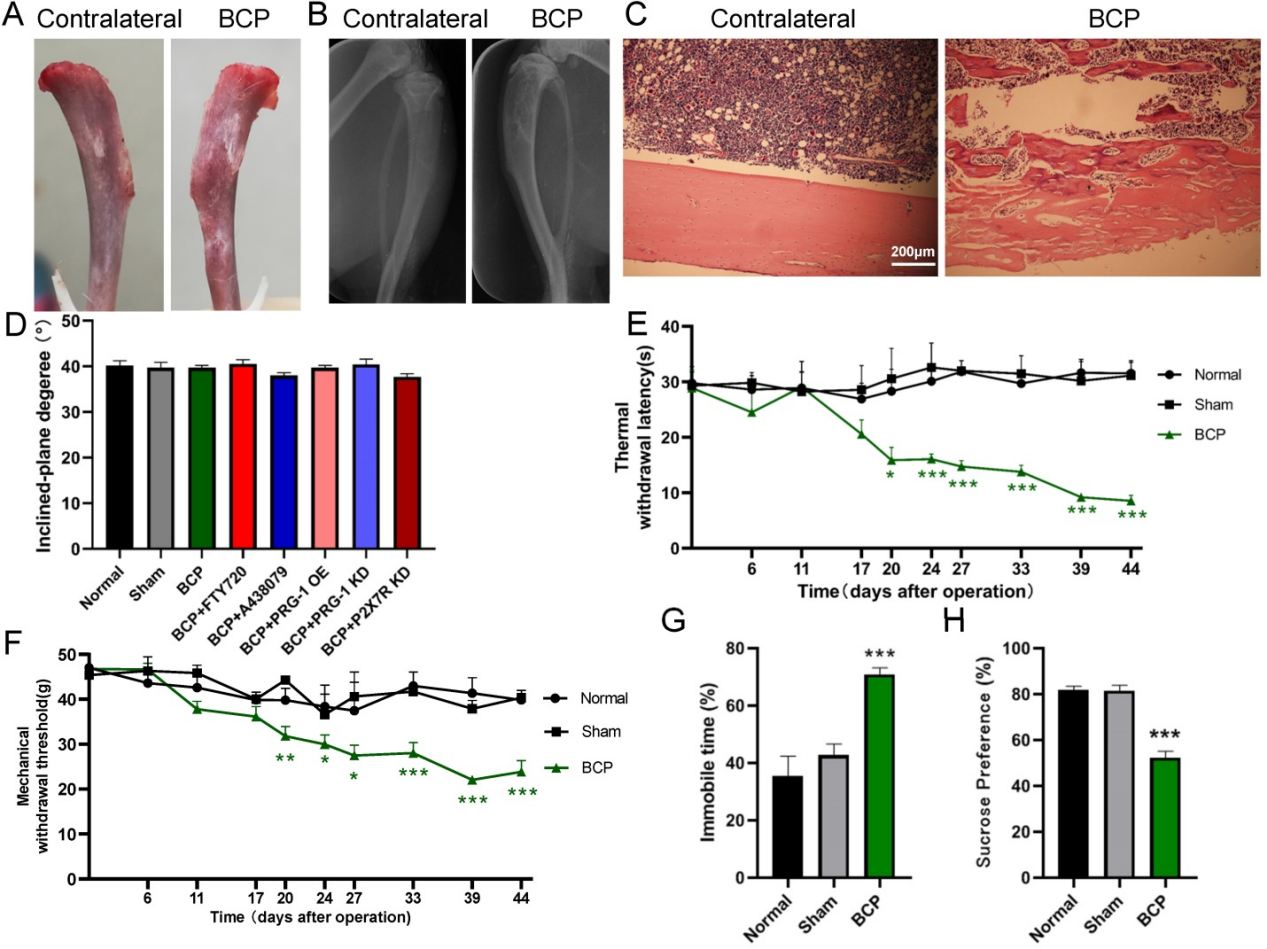
** Intra-tibia injection with SHZ-88 cells in SD rats caused bone destruction, thermal hyperalgesia, mechanical allodynia and depression-like behaviors. (A)** Photo of surface of dissected tibia of BCP operation (the rat tibia inoculated with SHZ-88 cells) and contralateral side on the postoperative day 28. **(B)** Radiograph of the cancer cell inoculation tibia and contralateral side in BCP rats. **(C)** Histopathological images of tibia sections stained by hematoxylin-eosin indicate obvious infiltrating osteolytic lesions of tibia. **(D)** Inclined plane test indicated that BCP, virus or drug microinjection didn't impair motor function of rats. n = 6; one-way ANOVA, *P* >0.05 among all groups. **(E)** The BCP rats showed significant decrease in TWL and produced thermal hyperalgesia on the operation-side hind paw from postoperative day 20 to 44. n = 12; one-way ANOVA, *: versus sham group at the same time point. **(F)** The BCP rats showed significant decrease in MWT and produced mechanical allodynia on the operation-side hind paw from postoperative day 20 to 44. one-way ANOVA, *: versus sham group at the same time point. **(G)** BCP rats showed prolonged immobility time in FST on postoperative day 20. n = 12; one-way ANOVA, *: versus sham group. **(H)** BCP group decreased sucrose preference on the sucrose preference test on postoperative day 20 compared with sham group. n = 12; one-way ANOVA, *: versus sham group. The data are expressed as the mean ± SEM; *P <0.05, **P <0.01 and ***P <0.001. BCP, bone cancer pain; TWL, thermal withdrawal latency; MWT, mechanical withdrawal threshold; FST, forced swimming test.

The BCP model was validated using radiological imaging and histological analysis. On day 28, X-ray picture of the rat tibia inoculated with SHZ-88 cells (BCP) showed discontinuity of the bone structure (Figure 1A), indicating bone destruction around the cancer cell injection site. After dissection, the surface of the BCP operation tibia was discontinuous and swelled up compared with the contralateral side (uninjured rat tibia) (Figure 1B). Hematoxylin-eosin staining of tibia sections showed marked infiltrating osteolytic lesions of tibia was detected in the proximal metaphysis and cavum medullare, such as tumor cells filling in the bone marrow cavity, bone destruction along the surfaces of trabecular, invasion and thinning of cortical bone, indicating the development of bone cancer in the tibia (Figure 1C). The deficits in the bone structure were insignificant on contralateral side tibia of BCP rats or in sham rats.

### Alterations of pain threshold values and depressive-like behaviors by cancer cell inoculation

Inclined plane test indicated that experimental procedures, such as BCP modeling operation, microinjection of virus or drugs didn't impair motor function of rats (1-way ANOVA, *P*>0.05; Figure 1D). The TWL and MWT on the ipsilateral side of the cancer cell inoculation, which was measured before BCP operation (day 0) and at days 6, 11, 17, 20, 24, 27, 33, 39 and 44 post-operation, was significantly decreased in BCP rats but not in sham rats from postoperative day 20 to 44 (1-way ANOVA, *P* <0.05 compared with sham group at the same time point, Figure 1E-1F), suggesting that thermal hyperalgesia and mechanical allodynia was developed in rats with BCP operation. In the forced swim test, BCP rats spent significantly longer time immobile than the sham rats did from postoperative day (POD) 20 (1-way ANOVA, *P*<0.001; Figure 1G). Moreover, BCP rats showed a decreased sucrose preference than sham rats from POD 20 (1-way ANOVA, *P* <0.001; Figure 1H). Overall, the above results suggested that BCP rats displayed clear comorbidity of bone cancer pain and depression.

### Hippocampal PRG-1 and P2X_7_ receptor are involved in bone cancer pain

**Figure 2:**
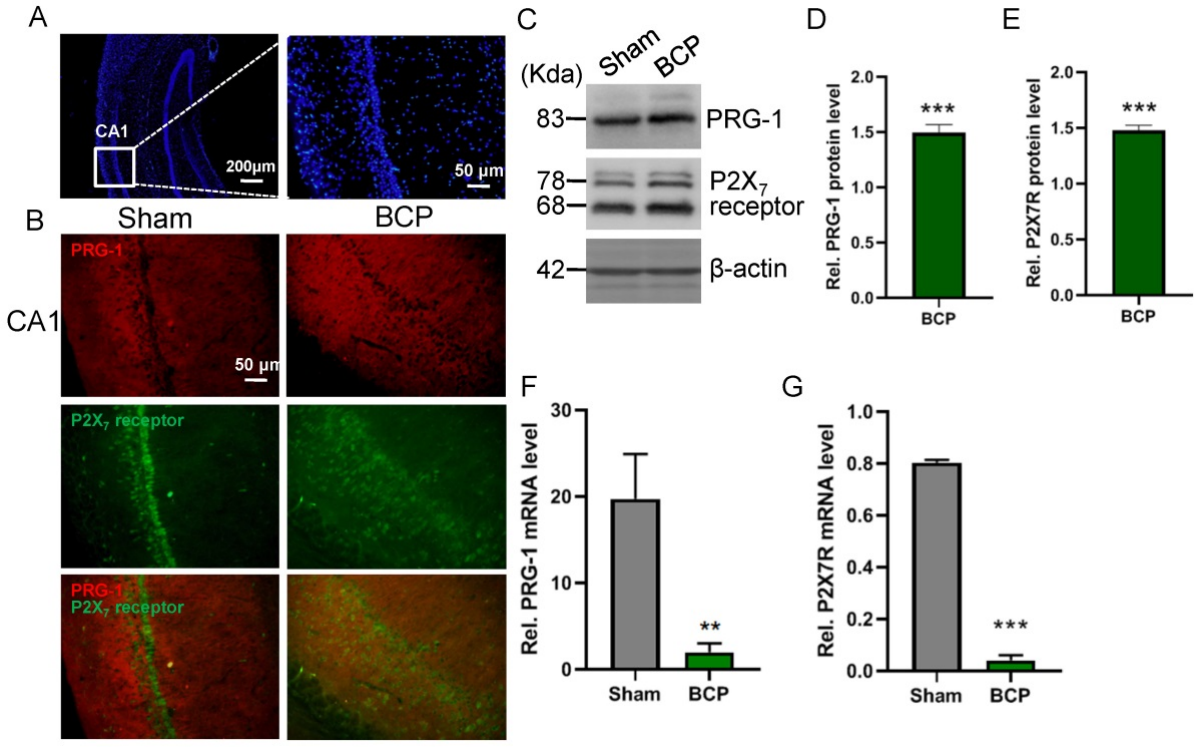
** Activation of hippocampal PRG-1 and P2X7 receptor in BCP rats. (A)** DAPI overview of a rat showing areas of CA1 (highlighted by white square). **(B)** Images of PRG-1 (red) and P2X_7_ receptor (green) in the hippocampus from sham and BCP rats by immunofluorescence on the POD 45 (scale bar = 50 μm). **(C)** Band of western blot and **(D-E)** quantitative analysis showed that the expression levels of PRG-1 and P2X_7_ receptor were increased in the hippocampus of BCP rats compared with corresponding sham group (n = 6, one sample t test). Values represent mean ± SEM. ***P < 0.001. **(F-G)** Quantitative analysis of qRT-PCR showed that the transcriptions of PRG-1 and P2X_7_ receptor mRNA were not increased in the hippocampus of BCP rats. β-actin was included as a control. The data are expressed as the mean ± SEM (n = 6). F: Mann-Whithnes-U test, G: unpaired Student t test, **P <0.01, ***P <0.001 compared to the sham group. BCP, bone cancer pain.

The expression levels of PRG-1 and P2X_7_ receptor in the hippocampus were both increased on postoperative day 45 in the BCP rats by immunofluorescence and western blot (Figure 2A-E), indicating activation of hippocampal PRG-1 and P2X_7_ receptor may be involved in bone cancer induced pain and depression. Furthermore, their subcellular distribution was studied by fluorescence microscopy, revealing partial colocalization between P2X_7_ receptor and PRG-1 (Figure 2B), suggesting a possible correlation of them. Meanwhile, transcripts for PRG-1 and P2X_7_ receptor were revealed in isolated hippocampus by qRT-PCR. Quantitative analysis showed that the transcriptions of PRG-1 and P2X_7_ receptor mRNA were not increased in the hippocampus of BCP rats (Figure 2F-G). These data suggested that the expression of PRG-1 and P2X_7_ receptor protein was specifically increased by translation, but not transcription step, in the BCP rats.

### BCP rats showed neuron deactivation and synaptic depression in the hippocampus

**Figure 3:**
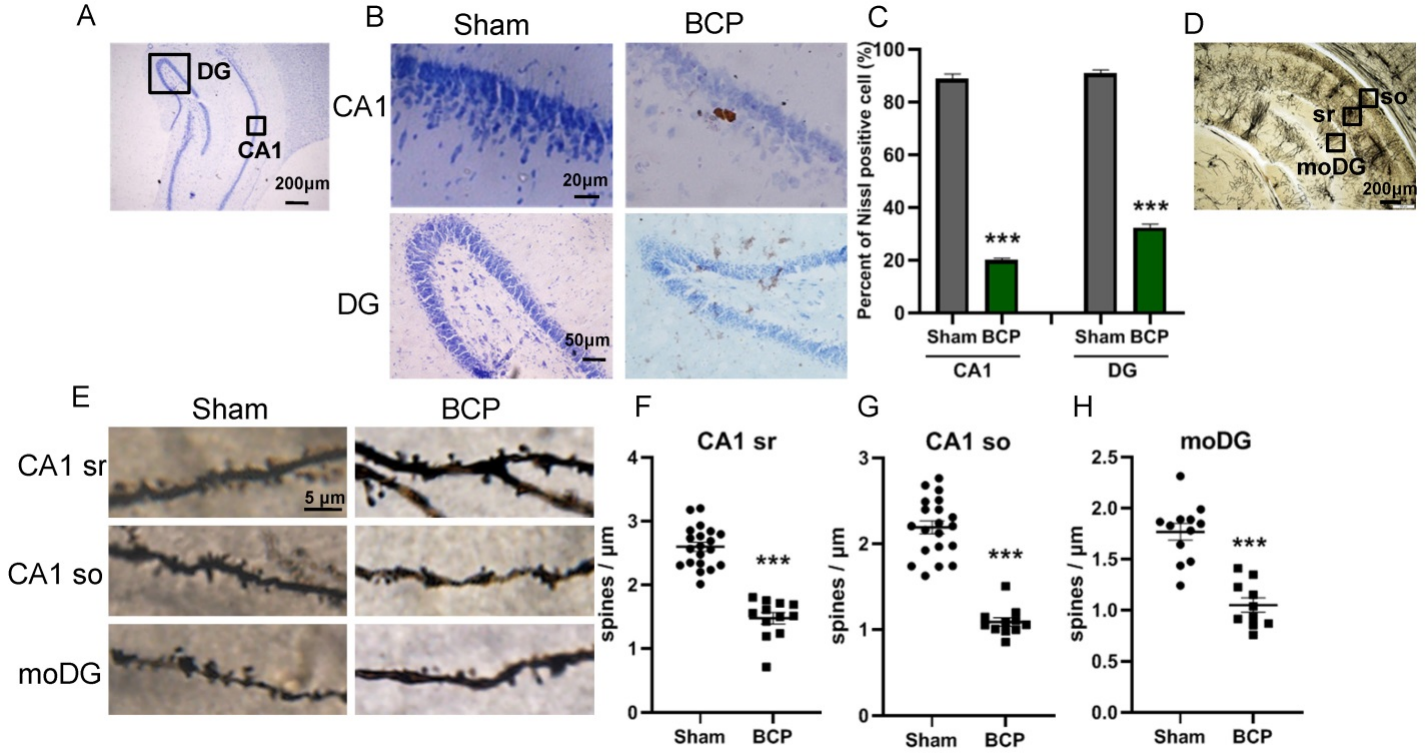
** BCP induced neuron deactivation and spine density decreased in the hippocampus. (A)** Nissl staining in whole hippocampus showing areas of CA1, DG zones. **(B)** Nissl staining in CA1, DG districts of rats' hippocampus. **(C)** The graph shows the percent of nissl positive cell. n = 6; student t test, ***P<0.001. **(D)** Hippocampus of a rat showing areas of spine assessment (highlighted by black squares): stratum radiatum (sr, apical dendrites) and stratumoriens (so, basal dendrites) of the CA1 region, and the stratum moleculare of the dentate gyrus (moDG). **(E)** Golgi-Cox staining and **(F-H)** quantitative analysis showed that the spine density was decreased in the hippocampus of BCP rats. F, n = 20 Sham and 12 BCP; G, n = 20 Sham and 11 BCP; H, n = 12 Sham and 10 BCP; n represents analyzed dendritic segments; student t test, ***P<0.001 compared to the sham group. The data are expressed as the mean ± SEM; BCP, bone cancer pain.

Since it has been proved that PRG-1 plays a critical role in synaptic plasticity 35, we next investigated the activation of neuron and dendritic spine in the hippocampus. The nissl staining, a method to examine histological change, was performed on the hippocampus. It was found that the hippocampus has normal histology in sham group, showing abundant nissl bodies in the cytoplasm of nerve cells and normal nucleoli in CA1 and DG in hippocampus. However, in the BCP hippocampus, degenerated nerve cells were found as indicated by the disappearance of nissl bodies and loss of nucleoli 45 days after BCP (Figure 3A-B). In CA1 and DG regions, the percent of nissl positive cell in the BCP group was lower than that in the sham group (Figure 3C). These data indicated that deactivation of neuron in the hippocampus was specifically induced in the BCP rats and may be related to the development of pain and depressive-like behavior in these rats.

To address the role of spine plasticity in bone cancer induced pain, we compared spine densities of sham and BCP groups on 45 days after operation *in vivo* by Golgi-Cox staining. Indeed, BCP group showed a significant reduction in spine density of about 30%-50% in hippocampus (CA1 sr, CA1 so and moDG regions) (Figure 3D-H), indicating a crucial role of spine density regulation by PRG-1 in bone cancer induced pain and depressive-like behavior in these rats.

### PRG-1 activation attenuated pain and depressive-like behaviors in BCP rats via PRG-1/PP2A signaling

**Figure 4:**
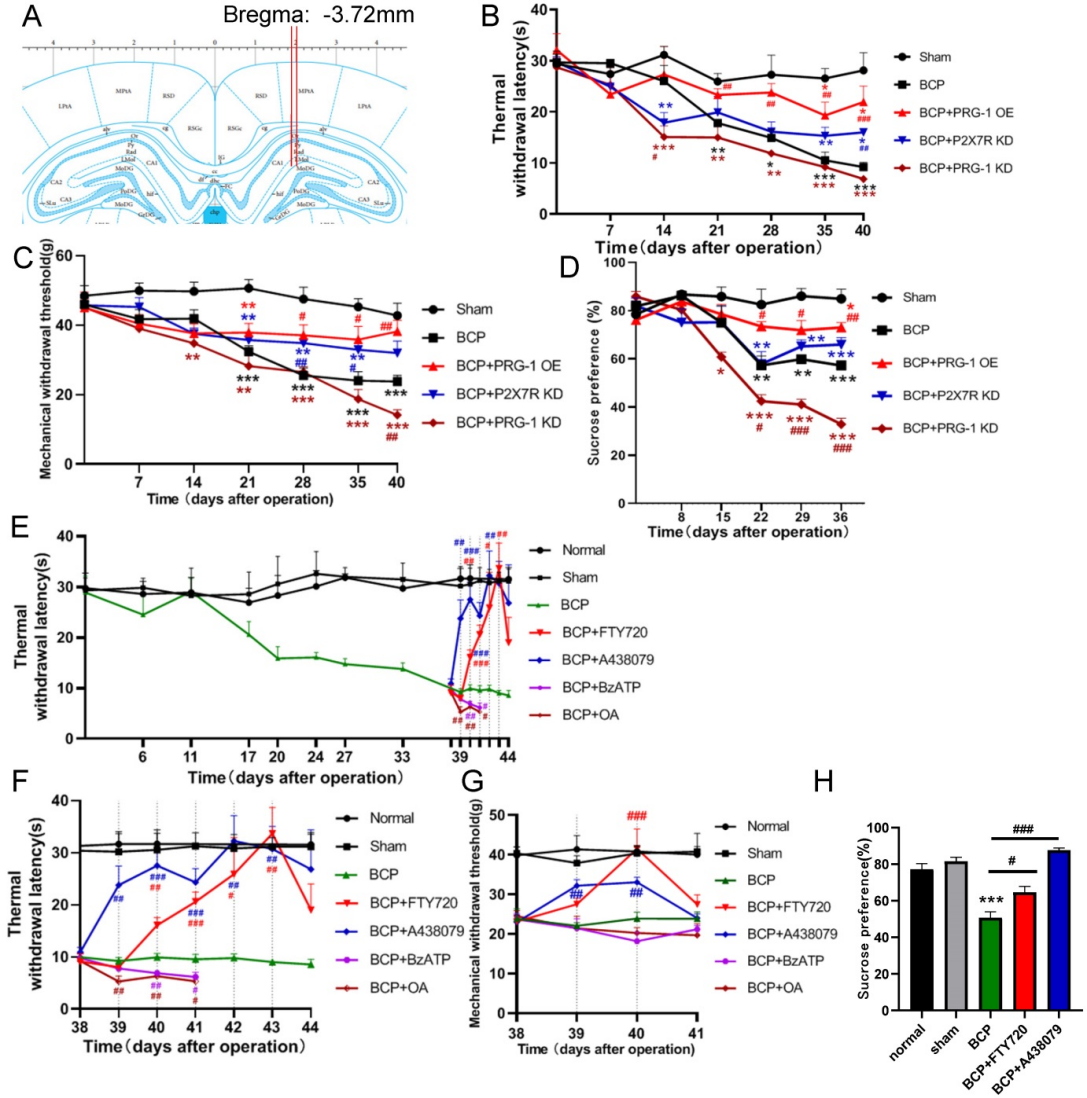
** PRG-1 attenuated pain and depression-like behaviors in BCP rats. (A)** Schematic diagram of hippocampal microinjection (x = ±2.0 mm, y = -3.72 mm, and z = -3.0 mm). **(B)** Hippocampus injection of virus vector LV-Plppr4 (PRG-1 OE) and LV-P2X7R-RNAi (P2X_7_ receptor KD) alleviated BCP-induced TWL decreases while LV-Plppr4-RNAi (PRG-1 KD) lead to early and intensified pain (n = 12; one-way ANOVA, *: versus Sham group at the same time point; #: versus BCP group at the same time point). **(C)** Hippocampus injection of virus vector LV-Plppr4 (PRG-1 OE) and LV-P2X7R-RNAi (P2X_7_ receptor KD) alleviated BCP-induced MWT decreases while LV-Plppr4-RNAi (PRG-1 KD) lead to early and intensified pain (n = 12; one-way ANOVA, *: versus Sham group at the same time point; #: versus BCP group at the same time point). **(D)** PRG-1 OE reversed the preference of sucrose (n = 12; one-way ANOVA, #: versus BCP group at the same time point). **(E)** Multiple FTY720 administration attenuated BCP-induced thermal withdrawal latency decreases from day 39 to 43 (n = 12; one-way ANOVA, *: versus Sham group at the same time point; #: versus BCP group at the same time point); dotted lines indicate pharmacological treatment). **(F)** Enlarged graph of D from POD 38 to 44. **(G)** Chronic FTY720 administration attenuated BCP-induced MWT decreases on day 40. (n = 6; Kruskal-Wallis test. #: versus BCP group at the same time point); dotted lines indicate pharmacological treatment). **(H)** FTY720 reversed the decline in the preference of sucrose consumption induced by BCP (n = 12 one-way ANOVA, *: versus Sham group at the same time point; #: versus BCP group at the same time point). The data are presented mean ± SEM. *P <0.05, **P <0.01, ***P <0.001; BCP, bone cancer pain; TWL, thermal withdrawal latency; MWT, mechanical withdrawal threshold; OE, overexpression; KD, knock down.

To test for the role of hippocampal PRG-1 and P2X_7_ receptor in bone cancer pain, the virus vector LV-Plppr4 (PRG-1 overexpression), LV-Plppr4-RNAi (PRG-1 knock down) or LV-P2X7R-RNAi (P2X_7_ receptor knock down) was slowly injected unilaterally into the hippocampus area one week before BCP operation (Figure 4A). PRG-1 overexpression has a role in alleviating pain, including thermal hyperalgesia and mechanical allodynia, and depression-like behavior, while PRG-1 KD leads to an early occurred and intensified pain and depression (Figure 4B-D), indicating the analgesic and antidepressant effect of PRG-1. P2X_7_ receptor KD also showed an analgesic effect in BCP rats, but not a depression-like behavior (Figure 4B-D), suggesting the algogenic effect of hippocampal P2X_7_ receptor.

PRG-1 regulates synaptic plasticity via intracellular PRG-1/PP2A signaling pathway 35. To test for the role of PRG-1/PP2A signaling in mediating PRG-1-dependent bone cancer induced pain, we preformed hippocampal microinjection of FTY720 (D39-43, 20 μg once per day), which serves as an activator of PP2A 76 and PRG-1/PP2A signaling 35, and BCP rats showed a significant increase in TWL and MWT with cumulative effect (Figure 4E-G) and also rescued sucrose preference of BCP rats (Figure 4H). In line with these results, selective inhibition of PP2A by okadaic acid (OA) 77 (D39-43, 100 nM, once per day) significantly decreased this TWL and MWT (Figure 4E-G). These data suggested that PRG-1/PP2A pathway attenuated bone cancer pain and depression-like behaviors in cancer-bearing rats. In contrast, selective inhibition of P2X_7_ receptor by antagonist A438079 (D39-43, 1 μg once per day) 78 also significantly increased TWL and MWT values without cumulative effect (Figure 4E-G). Moreover, A438079 were able to rescue sucrose preference of BCP rats (Figures 4H). In line with these results, selective enhancer of P2X_7_ receptor by BzATP 79 (D39-43, 100 nM once per day) significantly decreased this TWL (Figure 4E-F). These further verified the analgesic and antidepressant effect of PRG-1/PP2A pathway and the algogenic effect of hippocampal P2X7 receptor.

### PRG-1 rescued neuron deactivation and synaptic depression in the hippocampus of BCP rats

**Figure 5:**
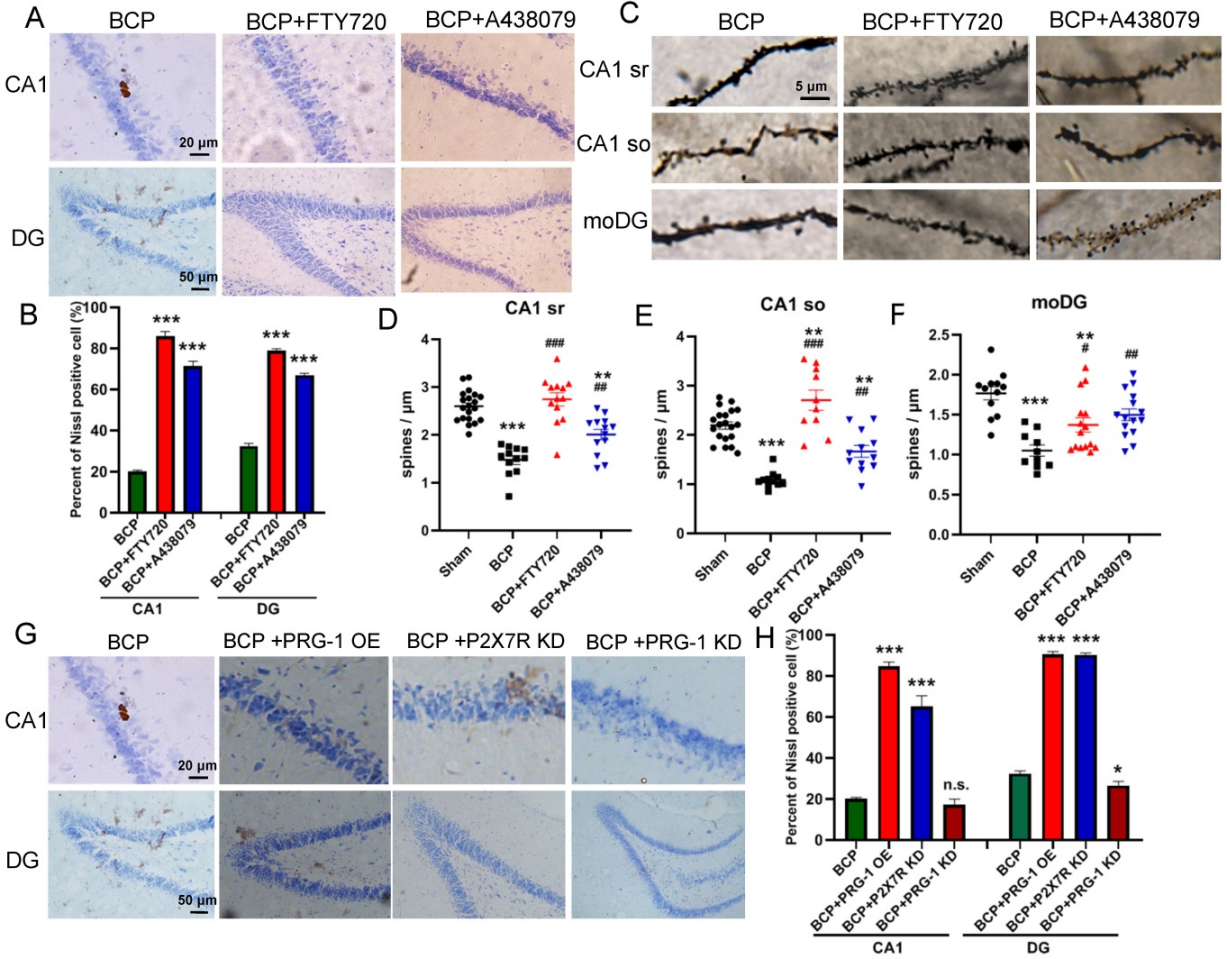
** PRG-1 attenuated neuron deactivation and synaptic depression in the hippocampus induced by BCP. (A)** Nissl staining in CA1, DG districts of rats' hippocampus under FTY720 or A438079 treatment. **(B)** The graph shows the percent of nissl positive cell. n = 6; one-way ANOVA, *: versus Sham group. **(C)** Images of Golgi-Cox staining and **(D-F)** quantitative analysis showed that the spine density depression induced by BCP was reversed by FTY720. (D, n =20 Sham, 12 BCP, 14 BCP+FTY720 and 13 BCP+A438079, one-way ANOVA; E, n = 20 Sham,11 BCP, 12 BCP+FTY720 and 12 BCP+A438079, one-way ANOVA; F, n = 12 Sham, 10 BCP, 15 BCP+FTY720 and 15 BCP+A438079, Kruskal-Wallis test; n represents analyzed dendritic segments; *: versus Sham group; #: versus BCP group); stratum radiatum (sr, apical dendrites) and stratum oriens (so, basal dendrites) of the CA1 region, and the stratum moleculare of the dentate gyrus (moDG). **(G)** PRG-1 OE and P2X_7_R KD rescued nissl bodies and nucleoli in CA1 and DG regions. The data are expressed as the mean ± SEM; *P<0.05, **P<0.01, ***P<0.001, BCP, bone cancer pain; OE, overexpression; KD, knock down.

The recovery of nerve cell morphology was observed in FTY720 treated group and PRG-1 overexpression group. We observed the re-appearance of nissl bodies and nucleoli in CA1 and DG regions (Figure 5A-B), indicating FTY720 promoted nerve regeneration as documented by nissl staining. Consistent with these results, BCP hippocampal microinjection of FTY720 showed an increase in spine density compared with untreated BCP rats in hippocampus CA1 (sr and so) and moDG regions (Figures 5C-F), further supporting specific PRG-1/PP2A interaction in spine formation and density induced by BCP. Meanwhile, treatment exclusive with A438079 enhanced nissl bodies and spine density in CA1 and moDG regions in BCP rats (Figures 5A-F). Furthermore, PRG-1 OE and P2X7R KD also rescued nissl bodies and nucleoli in CA1 and DG regions (Figure 5G-H). These indicated PRG-1 and PRG-1/PP2A pathway may play analgesic and antidepressant role by rescuing neuron deactivation and synaptic depression in the hippocampus of cancer-bearing rats.

### PRG-1/PP2A interaction were increased by hippocampal injection of FTY720

**Figure 6:**
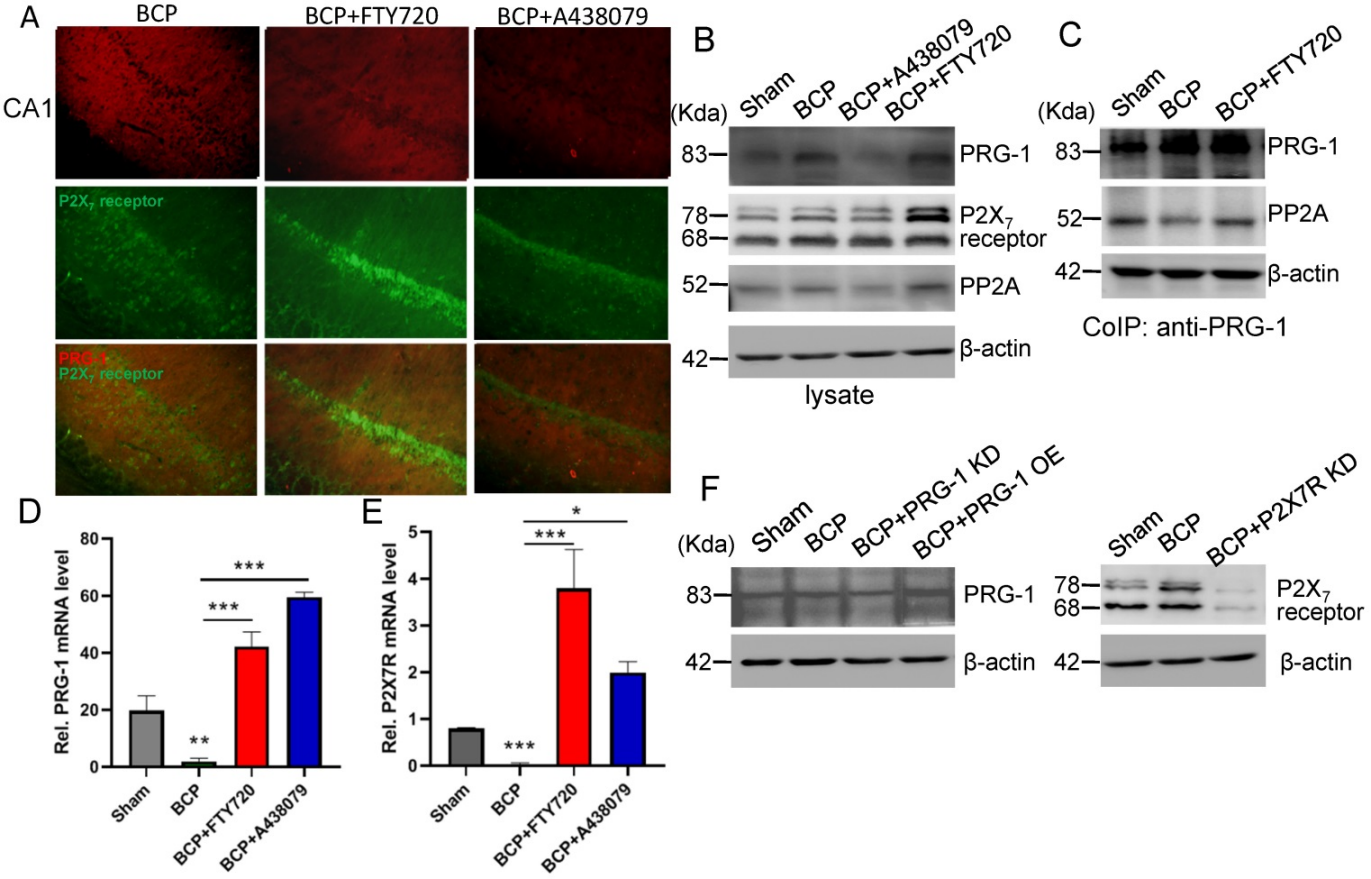
** PRG-1/PP2A interaction were increased by hippocampal injections of FTY720. (A)** Images of PRG-1 (red) and P2X_7_ receptor (green) in the hippocampus from BCP, BCP+FTY720 and BCP+A438079 rats by immunofluorescence on the POD 44 (scale bar = 50 μm). **(B)** Western blot showed that the expression of PRG-1 and P2X_7_ receptor was increased in the hippocampus of BCP+FTY720 rats. **(C)** Co-immunoprecipitation (IP) using a PRG-1 antibody shows PRG-1 and PP2A association. **(D-E)** Quantitative analysis of RT-qPCR showed that the transcription of PRG-1 and P2X7 receptor mRNA was increased in the hippocampus of BCP+FTY720 rats. β-actin was included as a control. The data are expressed as the mean ± SEM; n = 6; D: Kruskal-Wallis test, E: one-way ANOVA; *P <0.05, **P <0.005, ***P <0.001 compared to the BCP group. **(F)** Band of western blot showed the expression of PRG-1 and P2X_7_ receptor induced by virus expression vectors. BCP, bone cancer pain; OE, overexpression; KD, knock down; POD, postoperative day.

In hippocampus, the expression level of PRG-1 was further increased after FTY720 treatment (D39-43, 20 μg once per day) in the BCP rats by immunofluorescence and western blot analysis (Figure 6A-B), indicating the expression of PRG-1 is enhanced by FTY720 in BCP. Since our data so far provided strong evidence for a role of PRG-1 and PP2A in BCP, we assessed direct molecular interaction between both molecules. Using CoIP, we found that PRG-1/PP2A interaction was decreased in BCP rats but reversed by repeatly FTY720 treatments (Figure 6C), suggesting FTY720 relieved BCP and rescued neuron deactivation and synaptic anomalies via PRG-1/PP2A interaction. Furthermore, transcripts for PRG-1 and P2X_7_ receptor were revealed by quantitative RT-PCR. Quantitative analysis showed that the transcription of PRG-1 and P2X_7_ receptor mRNA was also increased in the hippocampus of BCP rats with FTY720 treatment (Figure 6D-E). These data suggested that the expression of PRG-1 and P2X_7_ receptor was specifically increased by transcription step in the BCP + FTY720 rats. Western blot showed the upregulated or silenced expression of PRG-1 and P2X_7_R induced by virus expression vectors (Figure 6F).

### PRG-1 relieves pain in BCP rats via P2X_7_R/PRG-1/PP2A signaling

**Figure 7:**
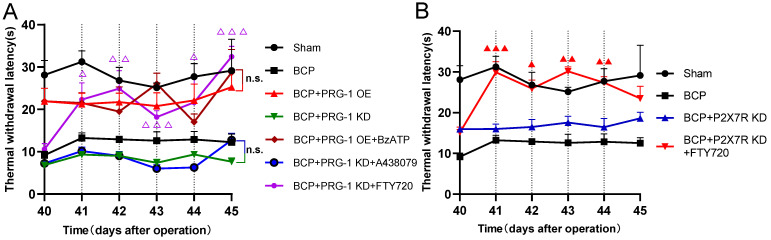
** PRG-1 relieves pain in BCP rats via P2X7R/PRG-1/PP2A signaling. (A)** TWL of drugs treatment under PRG-1 OE or KD (n = 6; Kruskal-Wallis test, △: versus BCP + PRG-1 KD group at the same time point). **(B)** FTY720 still reverses the TWL under P2X7R KD in BCP rats (n = 6; Kruskal-Wallis test, **▲**: versus BCP + P2X7R KD group at the same time point). The data are presented mean ± SEM. *P <0.05, **P <0.01, ***P <0.001; dotted lines indicate pharmacological treatment; BCP, bone cancer pain; TWL, thermal withdrawal latency; OE, overexpression; KD, knock down.

Here we identified hippocampal P2X_7_ receptor, PRG-1, PP2A are all involved in bone cancer pain and BCP induced depression, then we planned to determine the upstream and downstream relationship of them. Neither BzATP (D41-44, 100 nM, once per day), a P2X_7_ receptor agonist or A438079 (D41-44, 1 μg, once per day) could play an analgesic role under PRG-1 overexpression or PRG-1 KD in BCP rats, respectively (Figure 7A), indicating that P2X_7_ receptor play a role in BCP through PRG-1 and P2X_7_ receptor is the upstream signal of PRG-1 in BCP. Under PRG-1 KD, FTY720 (D41-44, 20 μg, once per day) remain an analgesic effect (Figure 7A), indicating that PP2A was downstream of PRG-1. Under P2X_7_R KD, FTY720 (D41-44, 20 μg, once per day) still played analgesic role (Figure 7B), indicating that PP2A was in the downstream of P2X_7_R. These results further verified that PRG-1 relieves pain in BCP rats via P2X_7_R/PRG-1/PP2A signaling.

## Discussion

Many studies suggests an involvement of PRG-1 in the pathogenesis and/or reparation attempts in neurological disease such as epilepsy 34, 38, 39, neurotrauma, Parkinson's disease 36, memory impairment 35 and cancer, including tumor progression or metastasis because of its putative target of LPA, a pro-cancerogenous factor 46, 80. These PRG-1 actions may be dependent of extracellular role in controlling LPA receptor-mediated excitatory synaptic transmission (or synaptic hyperexcitability) 34, 45, or in a cell-autonomous fashion in regulating spinogenesis via intracellular PRG-1/PP2A signaling pathway 35. In this study we report a new physiological role of PRG-1 for chronic pain and depressive-like behaviors. We demonstrate that BCP rats showed thermal hyperalgesia, depressive-like behaviors, reduced spine density, mild activation of PRG-1 and P2X7 receptor in hippocampus compared with sham group. But hippocampal PRG-1 and P2X_7_ receptor play contradictory roles in BCP. PRG-1 overexpression plays a role in alleviating pain and depression-like behaviors, while PRG-1 KD leads to an early-initiated intensified pain and depression, indicating activation of PRG-1 in the hippocampus participates in the analgesic mechanisms and antidepressant effect on bone cancer pain in rats. In order to alleviate the injury caused by bone cancer pain, PRG-1 expression in hippocampus was upregulated for a compensatory protective effect confront with the bone cancer induced pain, in accordance to the compensatory protective effect of PRG-1 on brain injury caused by flurothyl-induced recurrent neonatal seizures 81.

To uncover the analgesic mechanisms of PRG-1 on bone cancer pain in rats, we test for the role of PRG-1/PP2A signaling in mediating bone cancer induced pain. We used established pharmacological tools to stimulate PRG-1/PP2A signaling. Following hippocampal microinjection of FTY720, which serves as an activator of PP2A 76 and PRG-1/PP2A signaling 35, BCP rats showed a significant increase in TWL and MWT with cumulative effect and also rescued sucrose preference of BCP rats. In line with these results, selective inhibition of PP2A by okadaic acid 77 significantly decreased this TWL. These data suggested that PRG-1/PP2A pathway attenuated bone cancer pain and depression-like behaviors in cancer-bearing rats. These data provide evidence for the fact that PRG-1 relieves pain and depressive-like behaviors in rats with bone cancer pain by dendritic spine regulation in hippocampus via PRG-1/PP2A pathway.

In contrast, selective inhibition of P2X_7_ receptor by antagonist A438079 78 also significantly increased TWL and MWT without cumulative effect. Moreover, A438079 were able to rescue sucrose preference of BCP rats. In line with these results, selective enhancer of P2X_7_ receptor by BzATP 79 significantly decreased this TWL. P2X_7_ receptor KD also showed analgesic effect in BCP rats, but didn't change depression-like behavior. These further verified the algogenic effect of hippocampal P2X_7_ receptor on BCP.

The activation of spinal and supraspinal P2X_7_ receptors plays a key role in the occurrence and development mechanisms of various kinds of acute or chronic pain 54-56, including neuropathic pain and inflammatory pain 49, while the exact role of P2X_7_ receptor in BCP still remains controversial. Hansen et al. 58 discovered that P2X_7_ receptor knockout mice were more susceptible to BCP. Just the other way, Huang et al. 57 demonstrated that the activation of microglial P2X_7_ receptor in the rostral ventromedial medulla (RVM) is involved in the development of BCP by the mechanism of descending facilitatory via the spinal 5-HT. Li et al. 54 found that activation of the P2X_7_ receptor in the ventrolateral region of the periaqueductal gray (vlPAG) contributes to the analgesic effect of tramadol on bone cancer pain in rats. In this study, we verified the algogenic role of hippocampal P2X_7_ receptor on BCP in accordance with Shen et al. 82, who reported that activation of hippocampus P2X7 receptor aggravated neuropathic pain of diabetic rats.

Both PRG-1 and P2X_7_ receptor in hippocampus are involved in BCP and immunofluorescence revealed partial colocalization with P2X_7_ receptor of PRG-1. Meanwhile, we confirmed that P2X_7_ receptor plays a role in BCP through PRG-1, suggesting a possible correlation of PRG-1 and P2X_7_ receptor to jointly regulate the formation and maintenance of BCP, but there has not been any report on the relationship between P2X_7_ receptor and PRG-1 in hippocampus in pain modulation. However, immunoprecipitation studies failed to demonstrate a direct interaction between PRG-1 and P2X_7_ receptor. There could be some intermediary molecules between PRG-1 and P2X_7_ receptor in BCP regulation.

P2X_7_ receptor activation induced large numbers of Ca^2+^ influx activate a variety of downstream cell signal transduction pathways that excite sensory neurons, especially nociceptive neurons, thereby increasing their excitability and resulting in a higher level of pain sensation 59, 61, 62. Tokumitsu group identified a calmodulin-binding domain in the intracellular C terminus of PRG-1 (at aa 554-588), termed CBD, and suggest PRG-1 regulates excitatory synaptic transmission via PRG-1/CaM binding and controlled by changes in the concentration of intracellular Ca^2+^ 36. We previously reported that calmodulin binding domain of PRG-1 within the intracellular C-terminus mediates PP2A holoenzyme interaction. This PRG-1 CBD/PP2A interaction seems to be critical for organization of the adhesome 35. It is speculated that PP2A and CaM may be competitively bound to the intracellular CBD of PRG-1 in a Ca^2+^ -dependent manner.

**Figure 8:**
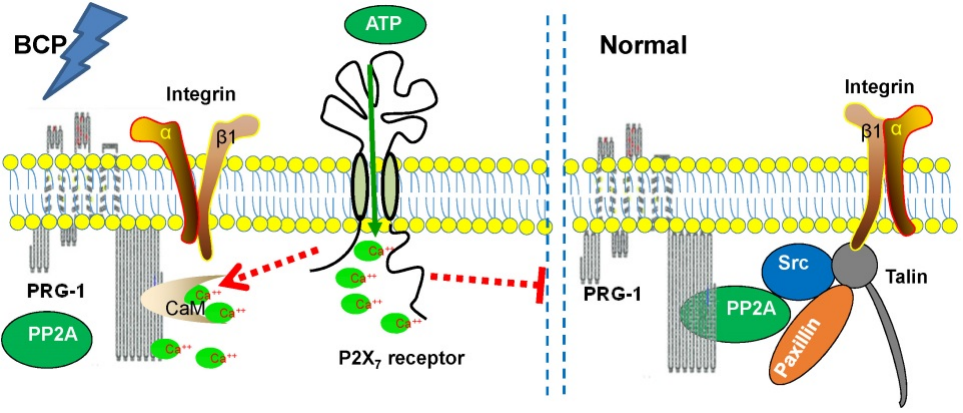
Schematic diagram of the signaling pathway analyzed.

Therefore, Ca^2+^ could be an intermediary molecule between PRG-1 and P2X_7_ receptor in BCP regulation. Based on this, we rationally hypothesized that after BCP operation, the hippocampal P2X_7_ receptor was activated, and further promote Ca^2+^ influx. As CaM/Ca^2+^ and PP2A binds to the same CBD region of PRG-1 C-terminal, the increase of intracellular Ca^2+^ changes PRG-1 function by inhibiting PRG-1/PP2A binding, leads to dendrite spine density decrease and bone cancer pain. In order to alleviate the injury caused by bone cancer pain, PRG-1 expression in hippocampus was upregulated for a compensatory protective effect confront with the bone cancer induced pain. If we treated BCP rats with FTY720 or PRG-1 overexpression, PRG-1 and PRG-1/PP2A binding were enhanced, counteract the effect of Ca^2+^ increase, enabled dendritic spine density recovery, alleviated bone cancer pain and depression. Thus, we hypothesized that a mechanism may exist in hippocampus in BCP rats, that is, P2X7 receptors in BCP rats are activated, and further promote Ca^2+^ influx, leading to PRG-1 function changing, ultimately inducing PRG-1 activation as a compensatory protective effect (Figure 8). The hypotheses need to be further validated.

Together with the functional and behavioral data, our molecular and morphological studies provide evidence to prove the fact that hippocampal PRG-1 drives a cell-autonomous signaling pathway involved in the regulation of spine density, and subsequently pain and depression-like behavior control via P2X_7_R/PRG-1/PP2A pathway, thus providing theoretical basis for a novel analgesia and depression relief strategies targeting PRG-1.

## Figures and Tables

**Table 1:** Primer sequences for qRT-PCR

Genes	Forward	Reverse
PRG-1	CGGATTCAGTTGCTATGACAGG	GGTCAGATCCCGAACAAATGTC
P2X7R	CCCTGTCCTATTTCGGTTTGG	CTGGTAGTTGAGACGGGAGGC
Actin	CCCCATTGAACACGGCATTAT	GCCACGCTCGGTGAGGATTTT

## Abbreviations

ATP: adenosine triphosphate
BCP: bone cancer pain
BSA: bovine serum albumin
CBD: calmodulin-binding domain
CoIP: Co-immunoprecipitation
DG: dentate gyri
EPSCs: excitatory postsynaptic currents
FST: forced swimming test
HE staining: Hematoxylin-eosin staining
HRPO: horseradish peroxidase
I.D.: inner diameter
i.p.: intraperitoneal
KD: knock down
LPA: lysophosphatidic acid
LPP: lipid phosphate phosphatase
LPPRs: lipid phosphate phosphatase-related proteins
moDG: molecular layer of dentate gyri
MWT: mechanical withdrawal threshold
OA: Okadaic Acid
O.D.: outside diameter
OE: overexpression
PFA: paraformaldehyde
PMSF: phenylmethanesulfonyl fluoride
POD: postoperative day
PP2A: protein phosphatase 2A
PRG-1: Plasticity-related gene 1
PRGs: plasticity-related genes
PVDF: polyvinylidene fluoride
qRT-PCR: quantitative reverse‐transcription polymerase chain reaction
RIPA: radioimmunoprecipitation assay
RTT: Rett syndrome
RVM: rostral ventromedial medulla
SDS-PAGE: sodium dodecyl sulfate-polyacrylamide gel electrophoresis
SEM: standard error of mean
so: stratum oriens
sr: stratum radiatum
TNFR1: tumor necrosis factor receptor 1
TWL: thermal withdrawal latency
vlPAG: ventrolateral region of the periaqueductal gray
WB: western blot
WT: wild type
